# Exploring the association between loneliness and breastfeeding duration in Chinese primiparas

**DOI:** 10.3389/fpsyt.2026.1919333

**Published:** 2026-10-14

**Authors:** Huiping Zhang, Pan Zeng, Miaomiao Chen, Rong Liu, Minjie Fu, Haijuan Wei, Siliang Chen, Haibin Wu

**Affiliations:** 1 Neonatal Intensive Care Unit, The Affiliated Children’s Hospital of Xi’an Jiaotong University, Xi’an, Shaanxi, China; 2 Xi’an Medical University, Xi’an, Shaanxi, China; 3 Xi’an Tus-Children’s Hospital, Xi’an, Shaanxi, China; 4 Science and Education Division, The Affiliated Children Hospital of Xi’an Jiaotong University, Xi’an, Shaanxi, China

**Keywords:** breast feeding, cortisol, loneliness, primiparity, prolactin

## Abstract

**Background:**

Reaching the recommendations for exclusive breastfeeding (EBF) during the first 6 months postpartum remains a worldwide challenge. Meanwhile, Chinese primiparas suffered from the loneliness associated with the traditional practice of zuoyuezi and the isolation during the COVID-19 pandemic. As far as we know, little is known about whether there is the association between loneliness in Chinese primiparas and breastfeeding duration.

**Purpose:**

This study aimed to explore whether loneliness in Chinese primiparas was associated with reduced breastfeeding duration, and further to explore the endocrine mechanisms.

**Methods:**

The Chinese primiparas were assessed for loneliness using the Los Angeles Loneliness Scale (R-UCLA) within 7–14 days postpartum. According to the scores of R-UCLA, the subjects were divided into three groups: no loneliness group (n=162), a low loneliness group (n=65) and a high loneliness group (n=40). The Postpartum Breastfeeding Questionnaire was filled out to record breastfeeding duration. The expression levels of the serum lactation hormone(prolactin)and the stress hormone (cortisol) were measured.

**Results:**

The time and rate of breastfeeding initiation within 3 days postpartum, as well as the frequencies of exclusive breastfeeding at 15 and 30 days postpartum, were significantly lower in Chinese primiparas with high loneliness; specifically, High loneliness was associated with lower exclusive breastfeeding rates by 25.2% (P<0.01) within 24 hours postpartum, 19.9% (*P* < 0.01) at 24 to 48 hours postpartum, 22.5% (*P* < 0.01) at 48 to 72 hours postpartum, 39.8% (*P* < 0.01) at 90 days postpartum, respectively. The probability of cumulative 90- and 180-day exclusive breastfeeding was the lowest in Chinese primiparas with high loneliness. The probability of cumulative 90- and 180-day exclusive breastfeeding rates decreased as the scores on the Loneliness Scale increased. Compared with primiparas in the no-loneliness and low-loneliness groups, those in the high-loneliness group exhibited the highest expression levels of the stress hormone (serum cortisol) and the lowest expression levels of the lactation hormone (serum prolactin) at 3, 15, and 45 days postpartum, respectively.

**Conclusion:**

Loneliness in Chinese primiparas during lactation was associated with lower exclusive breastfeeding rates and duration.

## 1 Introduction

Breastfeeding has numerous well-recognized beneficial effects on the lifelong health of both mother and child, including providing protection against breast cancer and type 2 diabetes, promoting optimal growth and development of infants and reducing the risk of allergic and infectious diseases in children (1). Exclusive breastfeeding (EBF) in the first 6 months postpartum is recommended by the United Nations Children’s Fund (UNICEF), the World Health Organization (WHO) (2), and the American Academy of Pediatrics. Despite global efforts, achieving the recommended exclusive breastfeeding (EBF) rates for the first six months postpartum remains a significant challenge (3),with current rates hovering around 40%. For instance, a cohort study conducted in Croatia revealed that the exclusive breastfeeding (EBF) rate was 39% at 3 months postpartum and only 8% at 6 months postpartum (4). Maintaining EBF is a major challenge for mothers, particularly for first-time mothers.

In China, there is a traditional postpartum practice called zuoyuezi, or “doing the month”(Pillsbury 1978). During ‘Zuoyuezi’ in China, the new mothers are expected to stay at home to restore their energy balance for at least one month after childbirth. To avoid physical contact with anything cold (e.g., cold winds, cold water, ice), going outdoors or parties and participation in social activities were forbidden, especially for Chinese primipara (5). To protect eyes, reading books, watching TV or movies and domestic duties were forbidden (5).The new mothers were usually taken care of by only one person, such as their mother-in-law, their own mother, or a paid caregiver. It is possible that social isolation or conflict with the mother-in-law during ‘Zuoyuezi’ could result in loneliness and affective disorders in Chinese primiparas (6). Ellen Y. Wan observed that among the 96% of women who underwent zuoyuezi, those who had their mother-in-law as the primary caregiver or who found zuoyuezi unhelpful were twice as likely to experience postpartum depression (7). More attention should be paid to the loneliness experienced by Chinese primiparas during ‘Zuoyuezi’. Furthermore, feelings of loneliness due to social isolation were prevalent among Chinese residents during the COVID-19 epidemic in 2020. Therefore, Chinese primiparas suffered from the loneliness associated with the traditional practice of zuoyuezi and the isolation during the COVID-19 pandemic.

Loneliness is a pervasive social problem all over the world. Loneliness is a negative emotional state which is associated with disappointing and unsatisfactory interpersonal relationships (8). Much attention had been paid to loneliness in the older adults (9). However, the loneliness in first-time mothers is often ignored and disregarded. Loneliness in the primipara should be paid more attention (10), which was associated with many physical and mental health problems. Breastfeeding duration was influenced by many factors, such as the mother’s age (11), race (12), level of education (13), presence or absence of obesity (14) and so on. As far as we know, little is known about the association between loneliness in primiparas and breastfeeding duration. Moreover, the mechanism through which loneliness influences breastfeeding duration remains unclear.

Loneliness may lead to the changes in stress and lactation hormones. Inconsistent results were reported about the association between loneliness and stress hormone (cortisol). The two studies indicated that there was no association between loneliness and awakening stress hormone (cortisol)levels or the diurnal cortisol slope (15). Another study showed that individuals with loneliness displayed a blunted cortisol rhythm (16). However, none of the above studies explored the association between loneliness and stress hormone expression levels in primiparas. Prolactin is a hormone secreted by the pituitary gland that promotes milk production. A decrease in lactation hormone (prolactin) could result in insufficient milk supply (17). Little is known about the association between loneliness and lactation hormone (prolactin) expression levels in primiparas.

In this study, we hypothesized that loneliness may lead to an increase in stress hormones (cortisol) and a decrease in lactation hormones (prolactin), thereby reducing the reduction of breastfeeding duration. We aimed to explore whether loneliness in Chinese primiparas was associated with reduced breastfeeding duration, to further to explore the endocrine mechanisms.

## 2 Method

This study was a bidirectional cohort design. Within 7–14 days postpartum, the three-item short 1980 version of the University of California, Los Angeles Loneliness Scale (R-UCLA) was used to assess loneliness in the subjects (18–20).According to the scores of R-UCLA, the subjects were divided into two groups: no loneliness group (n=176) and loneliness group (n=113). Based on the degree of loneliness, the subjects in the loneliness group were further divided into two subgroups: the low-loneliness group (n = 68) and the high-loneliness group (n=45). The reasons for selecting the timepoints (3, 15, and 45 days) are as follows: Day 3 is usually the day of hospital discharge; Day 15 marks the peak period for hospital visits due to breastfeeding difficulties; and Day 45 is when primiparas attend their first post-delivery follow-up visit. Maternal peripheral blood samples were collected on postpartum days 3, 15, and 45 to measure prolactin and cortisol levels.

### 2.1 Participants

The study subjects were primiparas and their newborns, recruited from January 2023 to February 2026. The inclusion criteria were as follows: 1. Aged 21–40 years; 2. First-time mother, full-term and singleton pregnancy; 3. Without any maternal medical illness, hypertension, diabetes mellitus or other severe diseases;4. Intention to breastfeed for at least 12 months. The exclusion criteria were as follows: the infants of the subjects showing the following signs: (1) infants with intrapartum complications; (2) preterm infants (< 37 weeks gestation); and (3) multiple births. A total of 267 subjects were recruited, and there were a no loneliness group (n=162), a low loneliness group (n=65) and a high loneliness group (n=40). Maternal and infant demographic data were obtained from the medical records. The investigation conformed to the Declaration of Helsinki and the protocol was conducted in accordance with Institutional Ethics Committee of the Affiliated Children’s Hospital of Xi’an Jiaotong University, Xi’an, Shaanxi, China. The ethics approval number is 20230301-3. Written informed consent was obtained from all subjects.

### 2.2 Experimental procedure

Main Demographic data of maternal and newborn infants were obtained from the medical records. Birth outcomes, including birth weight and gestation length, were obtained from the birth records. The Postpartum Breastfeeding Questionnaire was completed to record breastfeeding duration via telephone, WeChat, and interviews. The main items in the Postpartum Breastfeeding Questionnaire were as follows: the breastfeeding initiation time, the incidence rate of hypogalactia, the frequency of breastfeeding on the third day postpartum, as well as feeding practices observed at 15, 45, 90, and 180 days postpartum. The primiparas in both groups before and after delivery received the guidance on breastfeeding techniques and related lactation knowledge provided by the same groups of professional nurses. Additionally, they provided the individualized bedside breastfeeding guidance for all the primiparas. Thus, there were no differences in breastfeeding techniques. Meanwhile, the 24-item Hamilton Depression Rating Scale (HAMD) was used to assess the degree of maternal depression. The results indicated that there was no significant difference in maternal depression between the two groups of primiparas (**Supplementary Table 1**). Furthermore, there were no differences in demographic characteristics including maternal age, educational level, and family income between the two groups of primiparous women. During EBF, breast milk must be the infant’s only intake, without additional food or drink, including water. The primary outcome of this study was the proportion of exclusive breastfeeding at 90 and 180 days postpartum.

### 2.3 Loneliness assessment

Loneliness was assessed using the three-item short 1980 version of the University of California, Los Angeles Loneliness Scale (R-UCLA). The three items in the R-UCLA were as follows: 1. How often do you feel you lack companionship? 2. How often do you feel left out? 3. How often do you feel isolated from others? The method of scoring for each item was as follows: a score of 1 meant hardly ever or never, a score of 2 indicated some of the time and a score of 3 meant often. The range of the total score was 3-9. A score of 3 was regarded as no loneliness. A score of 4–6 and 7–9 indicated low loneliness and high loneliness, respectively.

### 2.4 Laboratory measurements

Peripheral blood samples of 3 ml were collected from the subjects. All the blood samples were collected within 30 minutes of awakening in the morning between 07:00 and 08:00 a.m. and in a fasting state. The levels of serum prolactin and cortisol were measured using Chemiluminescence Analysis (CLIA) and radioimmunoassay. All sample analyses were conducted by the chemistry laboratory at the Northwest Women and Children’s Hospital in Shaanxi Province. Laboratory personnel were blinded to mother and infant conditions. Serum cortisol was measured using a commercial Iodine [125I] Cortisol Radioimmunoassay kit from Chinese Beijing North Biotechnology Research Institute. Serum prolactin was measured using an RIA kit from Chinese Beijing North Biotechnology Research Institute. All samples were run in duplicate. Assay sensitivities were as follows: cortisol = 2 ng/ml, prolactin = 0.5μg/L. Intra-assay variability was <10% and inter-assay variability was <15%.

### 2.5 Data analysis

Data were collected and analyzed with SPSS 13.0 for Windows (SPSS Inc., Chicago, IL, USA). Quantitative variables were expressed as mean ± standard deviation. The Kolmogorov-Smirnov test was used to assess normal distribution of quantitative variables. Parametric values between the two groups were compared using an independent samples t-test or the Kolmogorov-Smirnov Z test, while non-parametric values were compared using the Mann-Whitney U test. Categorical variables were compared using Pearson’s chi-square test. Paired differences were compared using an independent samples t-test or Mann-Whitney U test and Kolmogorov-Smirnov Z test. Comparisons among multiple groups were analyzed using one-way analysis of variance (ANOVA) or the Kruskal-Wallis test. To further assess the estimated cumulative incidence of non-exclusive breastfeeding, Kaplan-Meier survival curves were performed with 3 groups segregated according to the scores of loneliness Scale. All probability values were two-tailed. A value of P < 0.05 was considered statistically significant.

## 3 Results

Among the recruited subjects, 22 were lost to follow-up over time, including 14 from the no loneliness group, 3 from the low loneliness group, and 5 from the high loneliness group. The retention rate was 92.4%. The reasons they were lost to follow-up were as follows: 1. The subjects did not wish to have a blood draw. 2. The subjects moved to another place to live or lost contact.

### 3.1 The association between loneliness and breastfeeding initiation

There were no significant differences in demographic characteristics of pregnant women in terms of maternal age, gestational age, residence, education, family income, professional status and matrimonial status (*P* > 0.05) (**Supplementary Table 1**). There were no significant differences in demographic characteristics of newborn infants (*P* > 0.05) (**Supplementary Table 2**). In the present study, we found that 39 percent of primiparas experienced loneliness. The time and rate of breastfeeding initiation were worse both in low loneliness group and high loneliness group within 3 days postpartum than that in no loneliness group (χ2 = 21.821, P = 0.001<0.05; χ2 = 8.434, P = 0.038<0.05). Furthermore, the time and rate of breastfeeding initiation were better in low loneliness group within 3 days postpartum than that in high loneliness group (χ2 = 8.256, P = 0.041<0.05). The incidence rate of hypogalactia in high loneliness group was higher than that of no loneliness group and low loneliness group at the 3rd day postpartum (χ2 = 8.658, P = 0.003<0.05; χ2 = 6.179, P = 0.013<0.05). However, there was no significant difference in the incidence rate of hypogalactia between the no-loneliness group and the low-loneliness group at the 3rd day postpartum (χ2 = 0.444, P = 0.505>0.05) (**Table 1**). The frequency of breastfeeding more than ten times per 24 hours in the high loneliness group was less than that of the no loneliness and low loneliness group at the 3rd day postpartum (χ2 = 10.646, P = 0.001<0.05; χ2 = 6.186, P = 0.013<0.05). However, there was no significant difference in the frequency of breastfeeding between the low loneliness group and no loneliness at the 3rd day postpartum (χ2 = 1.452, *P* = 0.022>0.05) (**Table 1**).

**Table 1:** The breastfeeding initiation time, the incidence rate of hypogalactia, and the frequency of breastfeeding on the third day postpartum.

Groups (n)	Breastfeeding initiation time			Incidence rate of hypogalactia n(%)	Frequency of breastfeeding (24h)	
	≤24h	24-48h	48-72h		≥10	<10
No loneliness group^1^(n=162)	61(37.7%)	85(52.4%)	4 (2.5%)	12(7.4%)	132(81.5%)	18(11.1%)
Low loneliness group ^3^(n=65) High loneliness group^2^(n=40) χ^2^ *P*	17(26.2%) 5(12.5%) 23.968 0.001	28(43.1%) 13(32.5%)	13(20.0%) 10(25.0%)	7(10.8%) 12(30.0%)	41(70.7%) 12(42.9%) 11.495 0.003	17(29.3%) 16(57.1%)

The breastfeeding initiation time.

1,2 χ^2^ = 8.434, *P* = 0.038<0.05; 1,3 χ^2^ = 21.821, *P* = 0.001<0.05; 2,3 χ^2^ = 8.256, *P* = 0.041<0.05.

The incidence rate of hypogalactia.

1,2 χ^2^ = 0.444, *P* = 0.505>0.05; 1,3 χ^2^ = 8.658, *P* = 0.003<0.05; 2,3 χ^2^ = 6.179, *P* = 0.013<0.05.

The frequency of breastfeeding on the third day postpartum.

1,2 χ^2^ = 1.452, *P* = 0.022>0.05; 1,3 χ^2^ = 10.646, *P* = 0.001<0.05; 2,3 χ^2^ = 6.186, *P* = 0.013<0.05.

### 3.2 The association between loneliness and breastfeeding duration

The frequencies of exclusive breastfeeding in the high loneliness group were worse than that of the no loneliness and low loneliness group at 15 days postpartum (χ^2^ = 19.060, *P* = 0.000<0.05; χ^2^ = 11.938, *P* = 0.003<0.05), at 45 days postpartum (χ^2^ = 14.733, *P* = 0.001<0.05; χ^2^ = 12.098, *P* = 0.002<0.05), at 90 days postpartum (χ^2^ = 15.878, *P* = 0.000<0.05; χ^2^ = 15.50, *P* = 0.000<0.05), respectively (**Table 2**). However, there was no significant difference in the duration of exclusive breastfeeding between the low loneliness group and no loneliness group at 15 days postpartum (χ^2^ = 2.524, *P* = 0.283>0.05), at 45 days postpartum (χ^2^ = 0.882, *P* = 0.643>0.05), at 90 days postpartum (χ^2^ = 0.521, *P* = 0.771>0.05), respectively (**Table 2**). High loneliness was associated with lower exclusive breastfeeding rates by 25.2% (*P* < 0.01) within 24 hours postpartum, 19.9% (*P* < 0.01) at 24 to 48 hours postpartum, 22.5% (*P* < 0.01) at 48 to 72 hours postpartum, 39.8% (*P* < 0.01) at 90 days postpartum, respectively.

**Table 2:** The feeding practices at 15,45,90 days postpartum.

Feeding practices	No loneliness group (n=162)	Loneliness	group (n=105)	χ^2^ *P value*
		Low (n=65)	High (n=40)	
At 15 days postpartum				21.839 0.000
exclusive breastfeeding	126(77.7%)	41(63.1%)	12(30.0%)	
combined feeding	28(17.3%)	17(26.2%)	16(40.0%)	
artificial feeding	8(5.0%)	7(10.8%)	12(30.0%)	
At 45 days postpartum				18.833 0.001
exclusive breastfeeding	109(67.3%)	38(58.5%)	10(25.0%)	
combined feeding	36(22.2%)	18(27.7%)	17(42.5%)	
artificial feeding	17(10.5%)	9(13.8%)	13(32.5%)	
At 90 days postpartum				21.792 0.000
exclusive breastfeeding	101(62.3%)	36(55.4%)	9(22.5%)	
combined feeding	40(24.7%)	19(29.2%)	12(30.00%)	
artificial feeding	21(13.0%)	10(15.4%)	19(47.5%)	

At 3 days postpartum: 1,2 χ^2^ = 2.524, *P* = 0.283>0.05; 1,3 χ^2^ = 19.060, *P* = 0.000<0.05; 2,3 χ^2^ = 11.938, *P* = 0.003<0.05.

At 15 days postpartum: 1,2 χ^2^ = 0.882, *P* = 0.643>0.05; 1,3 χ^2^ = 14.733, *P* = 0.001<0.05; 2,3 χ^2^ = 12.098, *P* = 0.002<0.05.

At 42 days postpartum: 1,2 χ^2^ = 0.521, *P* = 0.771>0.05; 1,3 χ^2^ = 15.878, *P* = 0.000<0.05; 2,3 χ^2^ = 15.50, *P* = 0.000<0.05.

Bold labels indicate time points postpartum.

### 3.3 The association between loneliness and cumulative 90- and 180-day non-exclusive breastfeeding

The Kaplan-Meier curves showed that the association between estimated cumulative incidence of non-exclusive breastfeeding and loneliness. When the subjects were divided into 3 groups according to the scores of loneliness Scale, the probability of cumulative 90- and 180-day non-exclusive breastfeeding both increased with the increase in the scores of loneliness Scale (**Figure 1**, **2**). The differences between the curves for the different groups were evaluated by log-rank test (*P* < 0.05 respectively). Although there was no significant difference in the duration of non-exclusive breastfeeding between the low loneliness group and no loneliness group at 90 days postpartum (*P* > 0.05), the probability of cumulative 180-day non-exclusive breastfeeding in Chinese primiparas with low loneliness was significantly higher than that in no loneliness group(*P* < 0.05).

**Figure 1:**
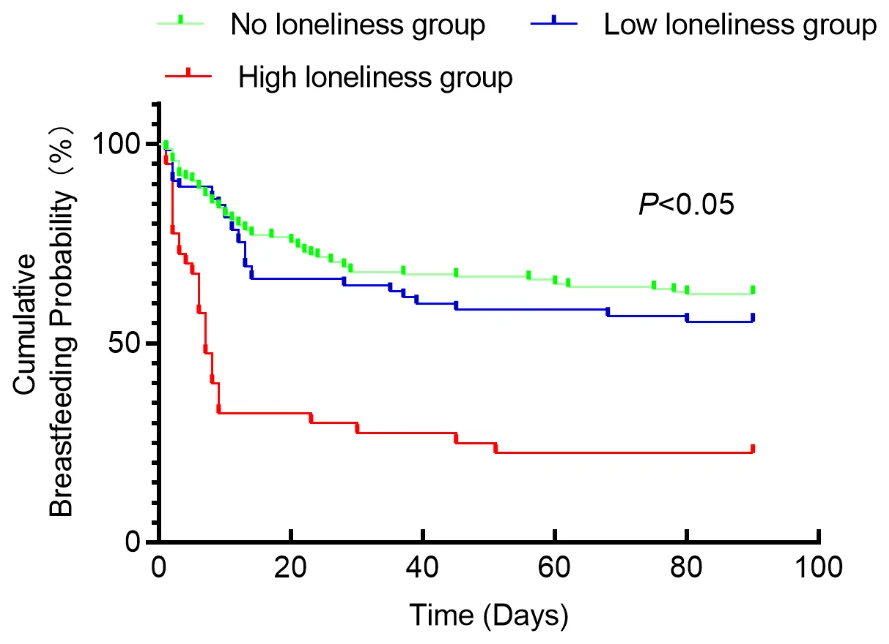
Cumulative 90-day non-exclusive breastfeeding according to the scores of loneliness Scale. Logrank *P* < 0.05 for no loneliness group vs. other groups.

**Figure 2:**
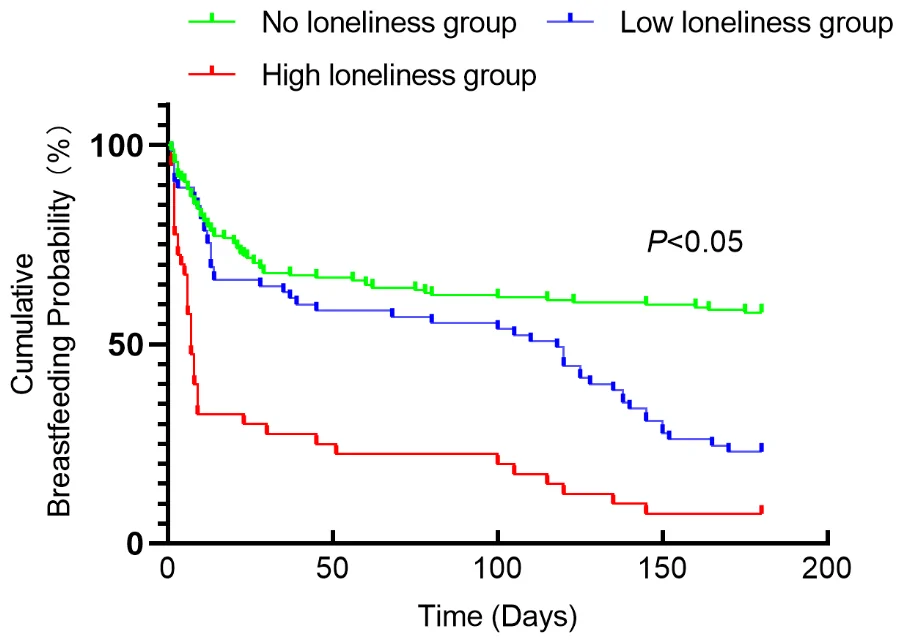
Cumulative 180-day non-exclusive breastfeeding according to the scores of loneliness Scale. Logrank *P* < 0.05 for no loneliness group vs. other groups.

### 3.4 The association between loneliness and serum prolactin concentrations in primiparas

Maternal serum prolactin concentrations were lower both in high loneliness group and low loneliness group than that in no loneliness at 3 days postpartum (321.18 ± 67.06μg/L vs 413.44 ± 94.26 μg/L, *P* < 0.05; 348.62 ± 36.99 μg/L vs 413.44 ± 94.26 μg/L, *P* < 0.05). Moreover, maternal serum prolactin concentrations in low loneliness group were higher at 3 days postpartum than that in high loneliness group (348.62 ± 36.99 μg/L vs 321.18 ± 67.06 μg/L, *P=0.039* < 0.05) (**Figure 3a**).

**Figure 3:**
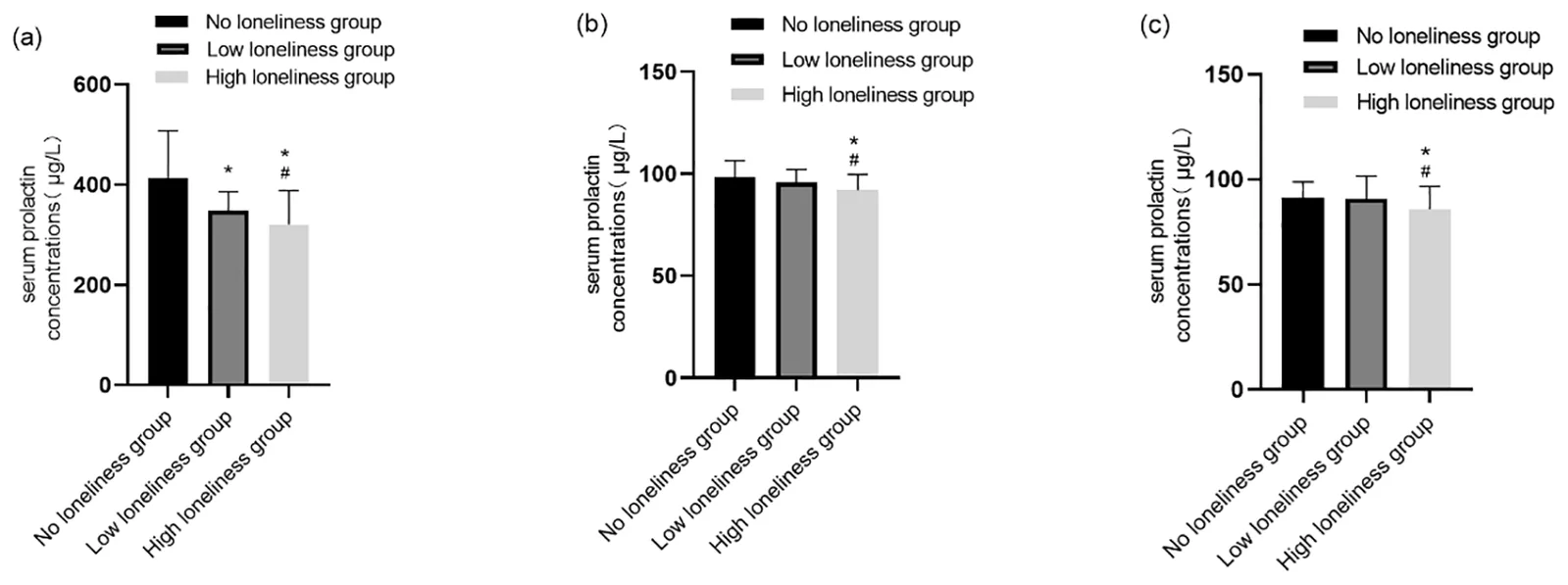
The association between loneliness and serum prolactin concentrations in primiparas. **(a)** at 3 days postpartum; **(b)** at 15 days postpartum; **(c)** at 45 days postpartum. ^*^P < 0.05 vs no loneliness group; ^#^ *P* < 0.05 vs low loneliness group.

Maternal serum prolactin concentrations in high loneliness group were lower than that of the low loneliness group and no loneliness group both at 15 day postpartum(92.00 ± 7.82μg/L vs 95.77 ± 6.43 μg/L, *P=0.012* < 0.05; 92.00 ± 7.82 μg/L vs 98.29 ± 8.19 μg/L, *P=0.001* < 0.05) and 45 day postpartum(85.92 ± 10.74μg/L vs 90.81 ± 10.73 μg/L, *P=0.016* < 0.05; 85.92 ± 10.74 μg/L vs 91.16 ± 7.67 μg/L, *P=0.02* < 0.05), respectively. However, there was no significant difference in maternal serum prolactin concentrations between the low loneliness group and no loneliness both at 15 days postpartum (95.77 ± 6.43 μg/L vs 98.29 ± 8.19 μg/L, *P=0.089*>0.05) and 45 day postpartum(90.81 ± 10.73 μg/L vs 91.16 ± 7.67 μg/L, *P=0. 86*>0.05), respectively (**Figures 3b, c**).

### 3.5 The association between loneliness and serum cortisol concentrations in primiparas

Maternal serum cortisol concentrations were higher both in high loneliness group and low loneliness group than that in no loneliness at 3 days postpartum (343.30 ± 41.24 ng/ml vs 247.27 ± 34.84 ng/ml, *P* < 0.05; 300.59 ± 25.23 ng/ml vs 247.27 ± 34.84 ng/ml, *P* < 0.05). Moreover, maternal serum cortisol concentrations in low loneliness group were lower at 3 days postpartum than that in high loneliness group (300.59 ± 25.23 ng/ml vs 343.30 ± 41.24 ng/ml, *P* < 0.05) (**Figure 4a**).

**Figure 4:**
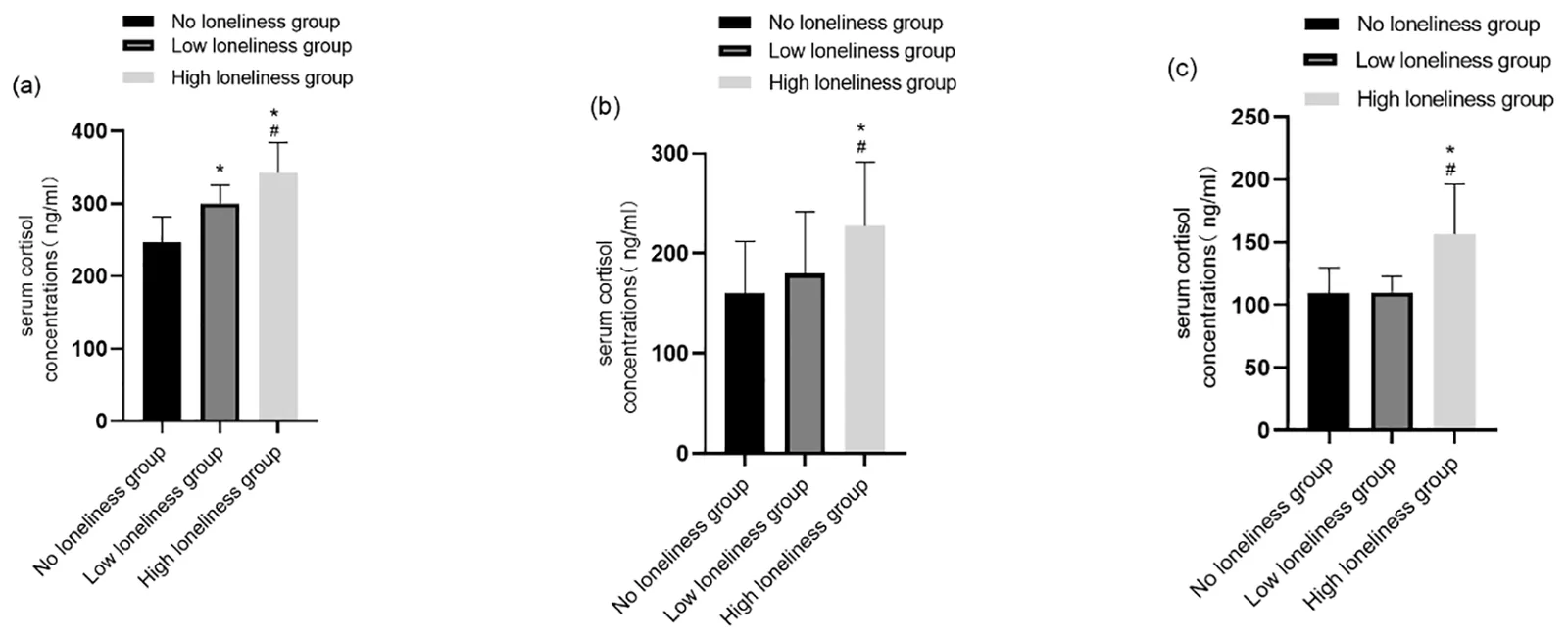
The association between loneliness and serum cortisol concentrations in primiparas. **(a)** at 3 days postpartum; **(b)** at 15 days postpartum; **(c)** at 45 days postpartum. ^*^P < 0.05 vs no loneliness group; ^#^ *P* < 0.05 vs low loneliness group.

Maternal serum cortisol concentrations in high loneliness group were higher than that of the low loneliness group and no loneliness both at 15 day postpartum (227.34 ± 64.14 ng/ml vs 179.59 ± 62.01 ng/ml, *P* < 0.05; 227.34 ± 64.14 ng/ml vs 159.88 ± 51.89 ng/ml, *P* < 0.05) and 45 day postpartum(156.47 ± 40.06 ng/ml vs 110.00 ± 12.79 ng/ml, *P* < 0.05; 156.47 ± 40.06 ng/ml vs 109.06 ± 20.66 ng/ml, *P* < 0.05), respectively. However, there was no significant difference in maternal serum cortisol concentrations between the low loneliness group and no loneliness group both at 15 day postpartum (179.59 ± 62.01 ng/ml vs 159.88 ± 51.89 ng/ml, *P=0.104*>0.05) and 45 day postpartum(110.00 ± 12.79 ng/ml vs 109.06 ± 20.66 ng/ml, *P=0.852*>0.05), respectively(**Figures 4b, c**).

## 4 Discussion

The present study is the first to address the association between loneliness in Chinese primiparas and breastfeeding duration. We found that the time and rate of breastfeeding initiation within 3 days postpartum and the frequencies of cumulative 90- and 180-day exclusive breastfeeding were the worst in Chinese primiparas with high loneliness. The probability of cumulative 90- and 180-day exclusive breastfeeding both decreased with increasing loneliness scale scores.

In this multicenter study, the primiparas came from five different cities located in Shaanxi Province in Northwest China and Shandong Province, East China. We found that 39 percent of primiparas experiencing loneliness. Although many studies have focused on loneliness in older adults, loneliness in first-time mothers is often overlooked. It has been reported women were more concerned with the interpersonal relationship and would like to creating good relationships, which may make them more vulnerable to the negative health effects of poor social connections (21). Women were significantly more likely to feel lonely and socially isolated than men (22). Furthermore, women are more sensitive to psychosocial stress (23). First-time mothers often experience intense pressure and psychosocial stress due to the need to care for their babies. Parental identity may be more important for mothers than fathers and for primiparous women than multiparous women (24, 25). Being a new mother entails significant responsibilities towards the child, which can make them feel helpless. The incidence of loneliness is higher in Chinese primiparas. The possible explanations are as follows. Firstly, in China, there is a traditional postpartum practice called ‘Zuoyuezi’, or “doing the month”. According to this custom, postpartum women are required to remain at home to recuperate for one month, during which they are prohibited from going out and must avoid exposure to wind and cold. This results strictly confines their physical activities to the domestic environment. Such mandatory isolation disrupts their social connections with the outside world, preventing them from participating in normal social activities. Most Chinese primiparous women, due to the superposition of childcare responsibilities and postpartum confinement taboos, have lives that revolve entirely around their infants, thereby forming the “motherhood cage” effect (26, 27). In contrast, Western postpartum recovery philosophies encourage early-stage outdoor activities (such as walking and community gatherings), thereby facilitating social connection (28, 29). During ‘Zuoyuezi’, the mothers-in-law or biological mothers of puerperae serve as primary caregivers, and among these families, conflicts arise from clashes between traditional taboos (e.g., prohibition of hair washing, enforcement of specific diets) and modern scientific parenting concepts (30).Within intergenerational power structures, postpartum women are compelled to compromise and suppress their genuine needs, which exacerbates emotional isolation. In Western nuclear family models, spouses serve as primary supporters, thereby reducing intergenerational intervention (31–33).Furthermore, feelings of loneliness due to social isolation were prevalent among Chinese residents affected by the COVID-19 epidemic in 2020. Therefore, more attention should be paid to the loneliness of primiparas. Given that breastfeeding benefits the lifelong health of both mother and child, it is widely recommended worldwide. The present study first focused on the effect of loneliness in Chinese primiparas on breastfeeding duration. We found that Chinese primiparas experiencing high levels of loneliness exhibited the longest delay and lowest rate of breastfeeding initiation within the first 3 days postpartum, along with the lowest rate of cumulative exclusive breastfeeding at 90 and 180 days. The probability of cumulative 90- and 180-day non-exclusive breastfeeding both increased with the increase in the scores of loneliness Scale. It is important that the effects of loneliness on breastfeeding are time dependent. Although there was no significant difference in the duration of exclusive breastfeeding between the low loneliness group and no loneliness at 90 days postpartum, the probability of cumulative 180-day exclusive breastfeeding in Chinese primiparas with low loneliness was significantly lower than that in no loneliness group. Therefore, loneliness may be an independent risk factor for non-exclusive breastfeeding. One possible explanation is that loneliness may disrupt maternal intentions regarding breastfeeding. Loneliness may impair mothers’ confidence and motivation for successful breastfeeding, leading to decreased possibility of initiation and maintenance of breast milk.

To further explore the endocrine mechanism of loneliness influencing breastfeeding, the expression levels of serum prolactin and cortisol were measured. Compared with the primiparas in no loneliness group and low loneliness group, there were the highest expression level of serum cortisol in the primiparas of high loneliness group at 3,15 and 45 day postpartum, respectively. Thus, loneliness was linked to an increased cortisol expression level. Furthermore, compared with the primiparas in no loneliness group and low loneliness group, the primiparas of high loneliness group displayed the lowest expression level of serum prolactin at 3,15 and 45 day postpartum, respectively. Numerous studies have shown that lower prolactin levels during lactation could result in a decrease of breast milk production or cessation of lactation. Additionally, the frequency of prolactin expression is correlated with 24-h milk volume. Therefore, we assume that reduced breastfeeding duration was associated with down-regulation of prolactin resulting from loneliness. One potential explanation for the down-regulation of prolactin in situations of loneliness is as follows. Prolactin is often regarded as a stress hormone because various stressors stimulate its secretion from the adenohypophysis into peripheral circulation (34). Increased prolactin levels are considered an adaptation to stress and attenuate stress circuitries (35). Thus, prolactin mediates the suppression of HPA axis hyperactivity following stress exposure, implying that prolactin inhibits the overexpression of cortisol. It has been reported that loneliness is associated with elevated cortisol levels. However, overexpression of glucocorticoids can inhibit the activity of the human prolactin (36). Therefore, cortisol overexpression in cases of loneliness could down-regulate prolactin levels.

This study innovatively put forward the hypothesis that loneliness in Chinese primiparas may lead to the reduction of breastfeeding duration based on Chinese primiparas suffering from the loneliness associated with the traditional practice of zuoyuezi and the isolation during the COVID-19 pandemi**c.** We advocate that government and community workers should pay more attention to the issue of loneliness among first-time mothers, thereby optimizing breastfeeding outcomes. We found that high loneliness was associated with lower exclusive breastfeeding rates. However, there was no significant difference in the duration of exclusive breastfeeding between the low loneliness group and no loneliness group, which may be related to the limited sample size. To explore the association between low loneliness scores and lower exclusive breastfeeding rates, the future research should aim to increase the sample size and conduct a multicenter clinical study.

## 5 Conclusions

Loneliness in Chinese primiparas during lactation was associated with lower exclusive breastfeeding rates and duration. We strongly recommend that government and community workers should pay more attention to the issue of loneliness among first-time mothers, thereby enhancing breastfeeding outcomes. Future studies should explore how to help first-time mothers in China alleviate loneliness, thereby increasing breastfeeding duration, for example through government and community-based public welfare activities, support groups for first-time mothers, and family support.

## Data Availability

The raw data supporting the conclusions of this article will be made available by the authors, without undue reservation.
