# Supplementary material for: Exploring the association between loneliness and breastfeeding duration in Chinese primiparas
Source: Front Psychiatry. 2026 Oct 14;17:1919333. doi: 10.3389/fpsyt.2026.1919333 (PMC13714646; doi:10.3389/fpsyt.2026.1919333)
Supplement: Supplementary file 1 [file Table1.docx]

**Table 1. Demographic characteristics in primiparas**

|  | No loneliness group (n=162) | Loneliness group (n=105) | | Values | *P* value |
| --- | --- | --- | --- | --- | --- |
| Age (y)（mean±SD） | 30.46±3.192 | 29.55±3.816 | | z=-1.714 | 0.086 |
| Gestational age (w)（mean±SD） | 38.592±0.979 | 38.958±1.061 | | t=1.798 | 0.075 |
| Residence |  |  | | χ^2^=0.777 | 0.378 |
| Rural area | 25(38.5%) | 12(30%) | |  |  |
| Urban area | 40(61.5%) | 28(70%) | |  |  |
| Education |  |  | | χ^2^=0.596 | 0.742 |
| Middle school | 13(20.0%) | 10(25.0%) | |  |  |
| High school | 18(27.7%) | 12(30.0%) | |  |  |
| Bachelor or higher 's degree | 34(52.3%) | 18(45.0%) | |  |  |
| Family income |  |  | | χ^2^=0.814 | 0.666 |
| <2000 RMB/m/person | 18(27.7%) | 8(20.0%) | |  |  |
| 2000–5000 RMB/m/person | 30(46.2%) | 21(52.5%) | |  |  |
| >5000Yuan/m/ person | 17(26.1%) | 11(27.5%) | |  |  |
| Professional status |  |  | | χ^2^=1.876 | 0.171 |
| Employed | 21(32.3%) | 8(20%) | |  |  |
| Unemployed | 44 (67.7%) | 32(80%) | |  |  |
| Matrimonial status |  |  | | χ^2^=1.418 | 0.234 |
| Married/cohabiting | 47(72.3%) | 33(82.5%) | |  |  |
| Single | 18(27.7%) | 7(17.5%) | |  |  |
| Maternal depression  The score of HAMD（mean±SD | 4.59±0.739 | 4.78±0.814 | | 1.173 | 0.243 |
| The score of HAMD（mean±SD） |  | Low loneliness group(n=65)  4.71±0.823 | high loneliness group(n=40)  4.85±0.791 | 0.847 | 0.899 |

**Table2. Demographic characteristics in newborn infants**

|  | No loneliness group (n=162) | Loneliness group (n=105) | Values | | | *P* value |  |
| --- | --- | --- | --- | --- | --- | --- | --- |
| Apgar index: 1st min（mean±SD） | 8.25±0.854 | 8.56±0.594 | z=-1.731 | | | 0.084 |  |
| Apgar index: 5th min（mean±SD） | 8.47±0.666 | 8.63±0.488 | z=-1.097 | | | 0.273 |  |
| Birth weight (g)（mean±SD） | 3236±417 | 3150±288 | z=-1.035 | | | 0.30 |  |
| Type of delivery |  |  | | χ^2^=0.569 | 0.451 | | |
| Vaginal delivery | 39(60%) | 21(52.50%) |  | | |  |  |
| Cesarean delivery | 26(40%) | 19(47.50%) |  | | |  |  |
| Gender |  |  | χ^2^=0.775 | | | 0.379 |  |
| Male | 35(53.8%) | 18(45.0%) |  | | |  |  |
| Female | 30(46.2%) | 22(55.0%) |  | | |  | |
